# Intake of dietary branched-chain amino acids reduces odds of metabolic syndrome: a cross-sectional study on the PERSIAN Kavar cohort study

**DOI:** 10.3389/fnut.2024.1403937

**Published:** 2024-10-17

**Authors:** Sara Shojaei-Zarghani, Mohammad Reza Fattahi, Zahra Mansourabadi, Ali Reza Safarpour

**Affiliations:** ^1^Colorectal Research Center, Shiraz University of Medical Sciences, Shiraz, Iran; ^2^Gastroenterohepatology Research Center, Shiraz University of Medical Sciences, Shiraz, Iran

**Keywords:** metabolic syndrome, hypertriglyceridemia, hyperglycemia, branched-chain amino acids, valine, leucine, isoleucine

## Abstract

**Background:**

Metabolic syndrome (MetS) is identified by the manifestation of a minimum of three out of five metabolic abnormalities, including insulin resistance, hypertension, hypertriglyceridemia, abdominal obesity, and low levels of high-density lipoprotein cholesterol. The present study aimed to assess the association between dietary branched-chain amino acids (BCAA) intakes and MetS, due to available conflicting evidence.

**Methods:**

A total of 4,860 individuals who had participated in the baseline phase of the PERSIAN (Prospective Epidemiological Research Studies in IrAN) Kavar cohort study were included in our study. The daily intake of valine, leucine, and isoleucine were evaluated using a semi-quantitative food frequency questionnaire. The association between dietary BCAA intake with MetS and its components was evaluated using logistic regression analysis.

**Results:**

The mean intake of BCAA among the included subjects was 7.65 (standard deviation [SD]: 2.92), and the prevalence of MetS was found to be 49.2%. Multivariable logistic regression analysis revealed an inverse association between 1-S.D. increment in dietary valine (odds ratio [OR] = 0.85, 95% confidence interval [CI]: 0.78–0.94), leucine (OR = 0.85, 95% CI: 0.77–0.93), isoleucine (OR = 0.84, 95% CI: 0.76–0.93), and total BCAA (OR = 0.85, 95% CI: 0.77–0.93) intake and the odds of MetS. There were also a significant association between BCAA intakes and hyperglycemia and hypertriglyceridemia.

**Conclusion:**

We observed a significant inverse association between dietary BCAA intake and MetS, hyperglycemia, and hypertriglyceridemia, regardless of confounding factors.

## 1 Introduction

Metabolic syndrome (MetS) is usually identified through the presence of at least three of five metabolic abnormalities, namely insulin resistance, hypertension, hypertriglyceridemia, abdominal obesity, and low levels of high-density lipoprotein cholesterol (HDL-C) (1). A recent report in 2022 has estimated the global prevalence of MetS to be between 12.5% and 31.4%, based on the modified Adult Treatment Panel III (ATP III) criteria (2). Given its high prevalence and significant association with mortality (3, 4), MetS remains an important public health concern. Prevention and management of MetS and its associated components heavily rely on lifestyle modifications, including dietary changes, weight control, and physical activity (5).

Branched-chain amino acids (BCAA) constitute a subset of essential amino acids that comprise valine (Val), leucine (Leu), and isoleucine (Ile). These amino acids play crucial roles in protein metabolism, mental health, and cognitive function, as evidenced by various studies (6). There is considerable controversy surrounding the association between BCAA intakes and risk of MetS and its components, with several studies not being derived from population-based samples. Some research indicates an inverse relationship between BCAA consumption and the risk of obesity (7, 8), diabetes (9), hypertension (10), and MetS (11), as well as a reduced risk of cardiovascular diseases (12). Conversely, some other studies have reported that higher dietary BCAA intake correlates with an increased risk of general obesity (13), diabetes (14), hypertension (15, 16), dyslipidemia (17), and MetS (18). Given these conflicting findings, the present study aims to clarify the potential association between BCAA intake and MetS, along with its individual components, by analyzing data from a large-scale population-based study conducted in Kavar County. This investigation aimed to enhance the understanding of the association by considering several confounders and examining the association based on the dietary source (plant-based or animal-based) of BCAAs.

## 2 Materials and methods

### 2.1 Study population

In the present cross-sectional study, we utilized baseline data from the PERSIAN Kavar Cohort Study (PKCS). The PKCS is a prospective population-based study initiated in 2017, comprising 4,997 individuals aged 35 to 70 residing in the urban area of Kavar County, Iran (19). All participants provided informed consent to participate in the PKCS, and the protocols were approved by the Ethics Committee of Shiraz University of Medical Sciences (Shiraz, Iran) (Code: IR.SUMS.REC.1402.061).

**FIGURE 1:**
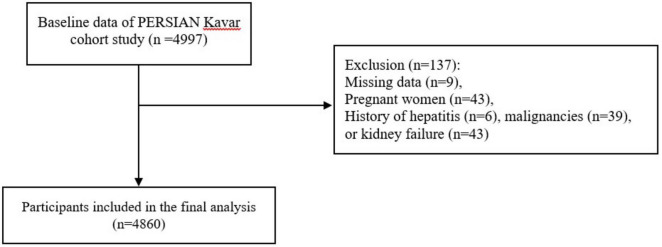
Flow diagram of the study.

For the current analysis, we excluded subjects with missing data (*n* = 9), as well as those who were pregnant (*n* = 43) or had kidney failure (*n* = 43), cancer (*n* = 39), or hepatitis (*n* = 6). None of the included participants were classified as heavy alcohol drinkers (defined as consuming more than 21 drinks per week for men and more than 14 drinks per week for women) (20). Additionally, all participants demonstrated plausible total energy intake, ranging from 800 to 8,000 kcal/day for men and from 600 to 6,000 kcal/day for women (21). Consequently, a total of 4,860 individuals were included in the final analysis (Figure 1).

### 2.2 Data collection

Sociodemographic information, including age, sex, education level, and ethnicity, as well as lifestyle habits (such as smoking, alcohol consumption, and physical activity), socioeconomic status (evaluated by the wealth score index), medical history, and medication use were gathered through face-to-face interviews using validated questionnaires. Trained personnel systematically collected anthropometric data, blood pressure measurements, and fasting venous blood samples using standardized procedures (19, 22). Commercial kits were used (Pars Azmoon, Iran) in combination with an auto-analyzer (model BT3000 Plus, Biotecnica^®^, Italy) to analyze the serum biochemical parameters.

The amount and frequency of food consumed in the past year were measured using a 113-item (plus five local foods) validated and semi-quantitative food frequency questionnaire (FFQ) administered by trained dietitians (23, 24). The nutrient contents of the foods were determined using the Food United States Department of Agriculture (USDA) Composition Tables (25). Iranian-native foods not listed in the USDA were equivalentized using the weighted average of their main ingredients (24). We estimated the intake of energy-adjusted Val, Leu and Ile using the residual method (26). Subsequently, we summed the newly generated variables to calculate the total energy-adjusted BCAAs intake.

### 2.3 Ascertainment of MetS

According to the “Joint Interim Statement of the International Diabetes Federation Task Force on Epidemiology and Prevention; National Heart, Lung, and Blood Institute; American Heart Association; World Heart Federation; International Atherosclerosis Society; and International Association for the Study of Obesity”, three or more of the following components was considered as MetS in individuals: waist circumference ≥ 90 cm for men and ≥ 80 cm for women (cut points in the Asian population); systolic blood pressure ≥ 130 mmHg, diastolic blood pressure ≥ 85 mmHg, or the use of antihypertensive medication; fasting plasma glucose (FPG) level ≥ 100 mg/dl or the use of anti-diabetic medication; HDL-C < 40 mg/dl for men and < 50 mg/dl for women, or the use of drugs for reduced HDL-C; and serum triglyceride (TG) level ≥ 150 mg/dl or the use of medication for elevated TG (1).

### 2.4 Statistical analysis

Data analysis was conducted using IBM SPSS version 25.0, with descriptive statistics (skewness, kurtosis, mean, and standard deviation [SD]) used to assess the normality of data distributions. Parametric data was expressed as mean ± SD, non-parametric variables were reported as median (range), and qualitative data was reported as frequency (percentages). Demographic, lifestyle, dietary, and biochemical characteristics were compared among subjects with and without MetS using independent sample t-test for parametric quantitative variables, Mann-Whitney U test for nonparametric variables, or chi-square test for qualitative data. Differences between quartiles of BCAA intake also were evaluated using the analysis of variance (ANOVA) test for parametric variables, the Kruskal–Wallis test for non-parametric parameters, and the Chi-square test for categorical variables. A multivariable logistic regression analysis was conducted to determine the independent association between BCAA intake and the likelihood of MetS and its components. Age, sex (male, female), ethnicity (Persian, Turk Nomad, others or mixed), education (illiterate, elementary, middle/high, college), socioeconomic status, smoking status (non-smoker, ex-smoker, current smoker), alcohol intake (yes, no), physical activity, body mass index (BMI), and dietary intakes of energy, saturated fatty acids, fiber, and protein were included in the fully-adjusted model according to the univariate analyses or the literature. A two-sided *P*-value < 0.05 was considered significant.

## 3 Results

Table 1 presents the characteristics of the subjects included in the study. The mean age of the total study population was 48.18 years (SD = 8.91). Out of the total population of 4,860, 2,392 (49.2%) were observed to exhibit MetS, with a considerably higher proportion of females (56.8%) than males (*P* < 0.001). Individuals with MetS had a greater tendency to be Persian, non-smokers, and non-drinkers, as well as to have lower educational levels and physical activity but higher BMI. They also had significantly lower dietary intakes of total energy (*P* = 0.034), saturated fatty acids (*P* = 0.002), and BCAA (*P* = 0.033) compared to others. Furthermore, subjects with MetS had significantly lower intakes of arginine (*P* = 0.031), cysteine (*P* = 0.004), phenylalanine (*P* = 0.014), proline (*P* = 0.015), serine (*P* = 0.010), threonine (*P* = 0.035), tyrosine (*P* = 0.040), and tryptophan (*P* = 0.018) (Supplementary Table 1).

**TABLE 1:** Characteristics of the included subjects according to the metabolic syndrome status.

Variable	All	With metabolic syndrome	Without metabolic syndrome	*P*-value
*n* (% of total)	4860	2392 (49.2)	2468 (50.8)	
**Sex, *n* (%)**				**<0.001**
Male	2386 (49.1)	1034 (43.2)	1352 (54.8)	
Female	2474 (50.9)	1358 (56.8)	1116 (45.2)	
**Education, *n* (%)**				**<0.001**
Illiterate	1509 (31.0)	877 (36.7)	632 (25.6)	
Elementary school	1505 (31.0)	720 (30.1)	785 (31.8)	
Middle and high school	1413 (29.1)	601 (25.1)	812 (32.9)	
College	433 (8.9)	194 (8.1)	239 (9.7)	
**Ethnicity, *n* (%)**				**<0.001**
Persian	3735 (76.9)	1907 (79.7)	1828 (74.1)	
Turk Nomad	943 (19.4)	395 (16.5)	548 (22.2)	
Others or mixed	182 (3.7)	90 (3.8)	92 (3.7)	
**Smoking, n (%)**				**<0.001**
Non-smoker	3723 (76.6)	1937 (81.0)	1786 (72.4)	
Ex-smoker	350 (7.2)	174 (7.3)	176 (7.1)	
Current smoker	787 (16.2)	281 (11.7)	506 (20.5)	
**Alcohol intake, *n* (%)**				**<0.001**
No	4430 (91.2)	2223 (92.9)	2207 (89.4)	
Yes	430 (8.8)	169 (7.1)	261 (10.6)	
**Wealth score index, *n* (%)**				0.756
1^st^ quartile	1215 (25.0)	583 (24.4)	632 (25.6)	
2^nd^ quartile	1220 (25.1)	611 (25.5)	609 (24.7)	
3^rd^ quartile	1394 (28.7)	686 (28.7)	708 (28.7)	
4^th^ quartile	1031 (21.2)	512 (21.4)	519 (21.0)	
Age (years), mean ± SD	48.2 ± 8.9	49.7 ± 9.0	46.7 ± 8.6	**<0.001**
BMI (kg/m^2^), mean ± SD	27.2 ± 4.8	29.2 ± 4.4	25.7 ± 4.6	**<0.001**
Waist circumference (cm), mean ± SD	95.9 ± 10.9	100.1 ± 9.4	91.8 ± 10.6	**<0.001**
Serum TC (mg/dl), mean ± SD	175.1 ± 37.0	179.8 ± 38.8	170.6 ± 34.6	**<0.001**
Serum HDL-C (mg/dl), mean ± SD	42.0 ± 9.4	38.8 ± 8.0	45.2 ± 9.7	**<0.001**
Serum LDL-C (mg/dl), mean ± SD	103.0 ± 30.6	102.7 ± 33.8	103.4 ± 28.4	0.433
Serum TG (mg/dl), median (range)	127.0 (993)	172.0 (971)	102.0 (827)	**<0.001**
FPG (mg/dl), median (range)	95.0 (366)	100.0 (365)	91.0 (363)	**<0.001**
SBP (mmHg), mean ± SD	118.1 ± 16.0	123.5 ± 16.8	112.8 ± 13.2	**<0.001**
DBP (mmHg), mean ± SD	76.7 ± 10.5	80.0 ± 10.8	73.6 ± 9.1	**<0.001**
Activity level (MET-h/week), mean ± SD	41.59 ± 6.5	41.1 ± 6.2	42.0 ± 6.8	**<0.001**
Dietary total energy intake (kcal/d), mean ± SD	2185.4 ± 608.2	2166.6 ± 603.6	2203.6 ± 612.1	**0.034**
Dietary total protein intake (g/d), mean ± SD	72.5 ± 22.1	72.3 ± 21.9	72.7 ± 22.4	0.585
Dietary SFA intake (g/day), mean ± SD	18.4 ± 7.5	18.0 ± 7.3	18.7 ± 7.7	**0.002**
Dietary fiber intake (g/day), mean ± SD	26.7 ± 9.6	27.0 ± 9.3	26.5 ± 9.9	0.067
Dietary valine intake (g/ day), mean ± SD	2.4 ± 0.9	2.3 ± 0.9	2.4 ± 0.9	**0.033**
Dietary leucine intake (g/ day), mean ± SD	3.3 ± 1.3	3.2 ± 1.2	3.3 ± 1.3	**0.026**
Dietary isoleucine intake (g/ day), mean ± SD	2.0 ± 0.8	1.9 ± 0.8	2.0 ± 0.8	**0.048**
Dietary BCAA intake (g/ day), mean ± SD	7.6 ± 2.9	7.6 ± 2.9	7.7 ± 2.9	**0.033**
Dietary animal-based BCAA intake (g/ day), mean ± SD	4.5 ± 2.2	4.5 ± 2.2	4.5 ± 2.2	0.254
Dietary plant-based BCAA intake (g/ day), mean ± SD	3.0 ± 1.1	2.9 ± 1.1	3.0 ± 1.1	**0.009**

BCAA, branched-chain amino acid; BMI, Body mass index; DBP, Diastolic blood pressure; FPG, Fasting plasma glucose; HDL-C, High-density lipoprotein cholesterol; LDL-C, Low-density lipoprotein cholesterol; SBP, Systolic blood pressure; SD, standard deviation; SFA, Saturated fatty acids; TC, total cholesterol; TG, triglyceride. Between-group differences were assessed using the independent sample *t*-test for parametric variables, the Mann–Whitney *U* test for non-parametric parameters, and the Chi-square test for categorical variables. *P* < 0.05 was considered significant. Animal-based BCAA included protein from meat, poultry, fish and tuna, eggs, dairy products, processed meat, and offal. Plant-based BCAA included protein from fruits, vegetables, grains, legumes, soy, and seeds. Bold denotes significant change.

The characteristics of the study participants, segregated according to the quartiles of the dietary energy-adjusted BCAA intake, are given in Table 2. The individuals in the highest quartile were more likely to be male, Persian, and highly educated with a considerable wealth score index level than those in the lowest quartile. Additionally, this group had significantly lower age, and physical activity levels but higher BMI, cholesterol, LDL-C levels, and fiber, saturated fatty acids, and protein intake compared to the lowest quartile. The intakes of Val (median: 31.7 mg/kg/day, minimum: 3.7, maximum: 115.5), Leu (median: 44.1 mg/kg/day, minimum: 4.5, maximum: 164.4), and Ile (median: 26.6 mg/kg/day, minimum: 2.8, maximum: 100.4) by the included subjects were within recommended dietary allowance (RDA) levels (27).

**TABLE 2:** Characteristics of the included subjects according to the quartiles of energy-adjusted BCAA intake.

Variables	Quartiles of energy-adjusted BCAA intake				*P*-value
	Q1	Q2	Q3	Q4	
*n*	1215	1215	1215	1215	
**Sex, *n* (%)**					**<0.001**
Male	608 (50.0)	559 (46.0)	562 (46.3)	657 (54.1)	
Female	607 (50.0)	656 (54.0)	653 (53.7)	558 (45.9)	
**Education, *n* (%)**					**<0.001**
Illiterate	537 (44.2)	396 (32.6)	329 (27.1)	247 (20.3)	
Elementary school	365 (30.0)	385 (31.7)	366 (30.1)	389 (32.0)	
Middle and high school	261 (21.5)	335 (27.6)	386 (31.8)	431 (35.5)	
College	52 (4.3)	99 (8.1)	134 (11.0)	148 (12.2)	
**Ethnicity, *n* (%)**					**<0.001**
Persian	839 (69.1)	927 (76.3)	985 (81.1)	984 (81.0)	
Turk Nomad	342 (28.1)	243 (20.0)	182 (15.0)	176 (14.5)	
Others or mixed	34 (2.8)	45 (3.7)	48 (4.0)	55 (4.5)	
**Smoking, *n* (%)**					**0.017**
Non-smoker	908 (74.7)	957 (78.8)	960 (79.0)	898 (73.9)	
Ex-smoker	91 (7.5)	79 (6.5)	76 (6.3)	104 (8.6)	
Current smoker	216 (17.8)	179 (14.7)	179 (14.7)	213 (17.5)	
**Alcohol intake, *n* (%)**					**<0.001**
No	1122 (92.3)	1142 (94.0)	1104 (90.9)	1062 (87.4)	
Yes	93 (7.7)	73 (6.0)	111 (9.1)	153 (12.6)	
**Wealth score index, *n* (%)**					**<0.001**
1^st^ quintile	494 (40.7)	331 (27.2)	210 (17.3)	180 (14.8)	
2^nd^ quintile	292 (24.0)	324 (26.7)	340 (28.0)	264 (21.7)	
3^rd^ quintile	281 (23.1)	334 (27.5)	379 (31.2)	400 (32.9)	
4^th^ quintile	148 (12.2)	226 (18.6)	286 (23.5)	371 (30.5)	
Age (years), mean ± SD	49.6 ± 9.0	48.6 ± 9.1	47.5 ± 8.7	46.9 ± 8.6	**<0.001**
BMI (kg/m^2^), mean ± SD	27.0 ± 4.9	27.4 ± 4.6	27.7 ± 4.9	27.7 ± 4.9	**0.001**
Waist circumference (cm), mean ± SD	95.2 ± 11.3	95.8 ± 10.3	96.6 ± 11.0	96.0 ± 10.8	**0.01**
Serum TC (mg/dl), mean ± SD	171.4 ± 37.2	174.5 ± 36.1	177.0 ± 37.1	177.5 ± 37.4	**<0.001**
Serum HDL-C (mg/dl), mean ± SD	41.7 ± 9.3	42.1 ± 9.7	42.2 ± 9.4	42.0 ± 9.3	0.521
Serum LDL-C (mg/dl), mean ± SD	99.7 ± 30.5	102.5 ± 30.3	104.6 ± 30.6	105.3 ± 30.7	**<0.001**
Serum TG (mg/dl), median (range)	125.0 (808)	125.0 (984)	130.0 (974)	129.0 (912)	0.440
FPG (mg/dl), median (range)	95.0 (366)	94.0 (281)	95.0 (293)	94.0 (291)	0.685
SBP (mmHg), mean ± SD	118.5 ± 16.7	118.3 ± 16.2	117.3 ± 15.1	118.3 ± 15.8	0.260
DBP (mmHg), mean ± SD	76.5 ± 10.8	76.6 ± 10.7	76.6 ± 10.1	77.3 ± 10.3	0.253
Activity level (MET-h/week), mean ± SD	42.7 ± 7.2	41.4 ± 6.2	41.1 ± 5.8	41.2 ± 6.7	**<0.001**
Dietary total energy intake (kcal/d), mean ± SD	2281.3 ± 656.7	2119.1 ± 569.1	2053.6 ± 525.6	2287.6 ± 638.3	**<0.001**
Dietary total protein intake (g/d), mean ± SD	69.0 ± 22.1	67.8 ± 19.5	68.97 ± 18.3	84.3 ± 23.9	**<0.001**
Percent of total protein intake (%),median (range)	8.3 (12.8)	10.0 (8.1)	11.2 (7.8)	12.4 (7.8)	**<0.001**
Dietary SFA intake (g/day), mean ± SD	17.2 ± 7.5	17.5 ± 7.0	17.6 ± 6.5	21.1 ± 8.2	**<0.001**
Dietary fiber intake (g/day), mean ± SD	26.8 ± 10.3	25.9 ± 9.2	25.5 ± 8.3	28.6 ± 10.3	**<0.001**
Dietary valine intake (g/ day), mean ± SD	1.7 ± 0.7	2.1 ± 0.6	2.4 ± 0.6	3.2 ± 0.9	**<0.001**
Dietary leucine intake (g/ day), mean ± SD	2.4 ± 0.9	2.9 ± 0.9	3.3 ± 0.8	4.5 ± 1.3	**<0.001**
Dietary isoleucine intake (g/ day), mean ± SD	1.4 ± 0.6	1.8 ± 0.5	2.0 ± 0.5	2.7 ± 0.8	**<0.001**
Dietary total BCAA intake (g/ day), mean ± SD	5.6 ± 2.1	6.8 ± 2.0	7.7 ± 1.9	10.5 ± 2.9	**<0.001**

BCAA, branched-chain amino acid; BMI, Body mass index; DBP, Diastolic blood pressure; FPG, Fasting plasma glucose; HDL-C, High-density lipoprotein cholesterol; LDL-C, Low-density lipoprotein cholesterol; SBP, Systolic blood pressure; SD, standard deviation; SFA, Saturated fatty acids; TC, total cholesterol; TG, triglyceride. Between-group differences in variables were assessed using the analysis of variance (ANOVA) test for parametric variables, the Kruskal–Wallis test for non-parametric parameters, and the Chi-square test for categorical variables. *P* < 0.05 was considered significant. Bold denotes significant change.

The ORs and 95% CIs for the odds of MetS based on dietary intake levels of BCAA are provided in Table 3. In the crude and adjusted models, there were no significant association between the quartiles of Val, Leu, Ile, and total BCAA intake and the odds of MetS. However, the fully adjusted model demonstrated a notable inverse linear association between 1-S.D. increment of dietary Val (OR = 0.85, 95% CI: 0.78–0.94, *P* = 0.001), Leu (OR = 0.85, 95% CI: 0.77–0.93, *P* = 0.001), Ile (OR = 0.84, 95% CI: 0.76–0.93), and total BCAA (OR = 0.85, 95% CI: 0.77–0.93) intake and the odds of MetS. Moreover, in an additional analysis, no significant association was identified between the ratio of animal-based to plant-based BCAA intake and the likelihood of MetS (OR per 1-S.D. increase = 1.05, 95% CI: 0.95–1.17, *P* = 0.300).

**TABLE 3:** Odds of metabolic syndrome according to the quartiles of energy-adjusted valine, leucine, isoleucine, and total BCAA intake.

Models	Quartile of energy-adjusted BCAA intake				BCAA intake per 1-S.D.	*P*
	Q1	Q2	Q3	Q4		
**Energy-adjusted valine, median (range)**	1.32 (3.91)	2.05 (0.58)	2.63 (0.62)	3.42 (5.46)		
Event/Total	598/1215	595/1215	601/1215	498/1215		
OR (95%CI) *	1.00 (Ref.)	0.99 (0.84–1.16)	1.01 (0.86–1.18)	1.00 (0.85–1.17)	0.98 (0.92–1.03)	0.406
OR (95%CI) ^†^	1.00 (Ref.)	0.96 (0.80–1.15)	0.99 (0.83–1.20)	1.03 (0.85–1.25)	0.98 (0.91–1.05)	0.611
OR (95%CI) ^‡^	1.00 (Ref.)	0.90 (0.75–1.08)	0.90 (0.74–1.10)	0.83 (0.66–1.05)	0.85 (0.78–0.94)	**0.001**
**Energy-adjusted leucine, median (range)**	2.26 (3.88)	2.98 (0.58)	3.55 (0.62)	4.34 (6.04)		
Event/Total	595/1214	600/1216	606/1215	591/1215		
OR (95%CI) *	1.00 (Ref.)	1.01 (0.86–1.19)	1.03 (0.88–1.21)	0.98 (0.84–1.15)	0.97 (0.92–1.03)	0.327
OR (95%CI) ^†^	1.00 (Ref.)	1.00 (0.83–1.20)	1.03 (0.85–1.24)	1.03 (0.85–1.25)	0.98 (0.92–1.05)	0.651
OR (95%CI) ^‡^	1.00 (Ref.)	0.93 (0.78–1.12)	0.92 (0.75–1.12)	0.82 (0.65–1.04)	0.85 (0.77–0.93)	**0.001**
**Energy-adjusted isoleucine, median (range)**	0.95 (4.00)	1.68 (0.59)	2.23 (0.61)	3.03 (5.74)		
Event/Total	594/1215	594/1214	600/1216	604/1215		
OR (95%CI) *	1.00 (Ref.)	1.00 (0.85–1.17)	1.02 (0.87–1.19)	1.03 (0.88–1.21)	0.98 (0.93–1.04)	0.520
OR (95%CI) ^†^	1.00 (Ref.)	0.99 (0.83–1.18)	1.01 (0.84–1.22)	1.07 (0.88–1.29)	0.98 (0.92–1.06)	0.675
OR (95%CI) ^‡^	1.00 (Ref.)	0.93 (0.77–1.12)	0.90 (0.74–1.11)	0.86 (0.68–1.09)	0.84 (0.76–0.93)	**0.001**
**Energy-adjusted BCAA, median (range)**	4.53 (11.80)	6.70 (1.74)	8.40 (1.84)	10.79 (17.26)		
Event/Total	596/1215	596/1215	605/1215	595/1215		
OR (95%CI) *	1.00 (Ref.)	1.00 (0.85–1.17)	1.03 (0.88–1.21)	0.99 (0.85–1.17)	0.98 (0.92–1.03)	0.412
OR (95%CI) ^†^	1.00 (Ref.)	1.00 (0.83–1.20)	1.02 (0.85–1.24)	1.04 (0.85–1.26)	0.98 (0.92–1.05)	0.645
OR (95%CI) ^‡^	1.00 (Ref.)	0.94 (0.78–1.13)	0.91 (0.75–1.12)	0.83 (0.65–1.05)	0.85 (0.77–0.93)	**0.001**
**Animal to plant-based BCAA ratio, median (range)**	−0.30 (3436.05)	0.80 (0.69)	1.61 (1.20)	4.07 (1209.57)		
Event/Total	575/1215	567/1215	638/1215	612/1215		
OR (95%CI) *	1.00 (Ref.)	0.97 (0.83–1.14)	1.23 (1.05–1.44)	1.13 (0.96–1.32)	1.08 (0.98–1.19)	0.120
OR (95%CI) ^†^	1.00 (Ref.)	0.93 (0.77–1.11)	1.14 (0.95–1.37)	1.13 (0.94–1.36)	1.06 (0.95–1.18)	0.274
OR (95%CI) ^‡^	1.00 (Ref.)	0.91 (0.76–1.10)	1.09 (0.91–1.32)	1.07 (0.88–1.29)	1.05 (0.95–1.17)	0.300

BCAA, branched-chain amino acids; CI, confidence interval; OR, odds ratio. ORs and 95% CI were determined by multivariable logistic regression.

* Model 1: Crude and unadjusted,

^†^ Model 2: adjusted for age, sex, ethnicity, education, socioeconomic status, smoking status, alcohol intake, physical activity, body mass index, energy, saturated fatty acids, and fiber intakes,

^‡^ Model 3: additionally, adjusted for protein. Bold denotes significant change.

**FIGURE 2:**
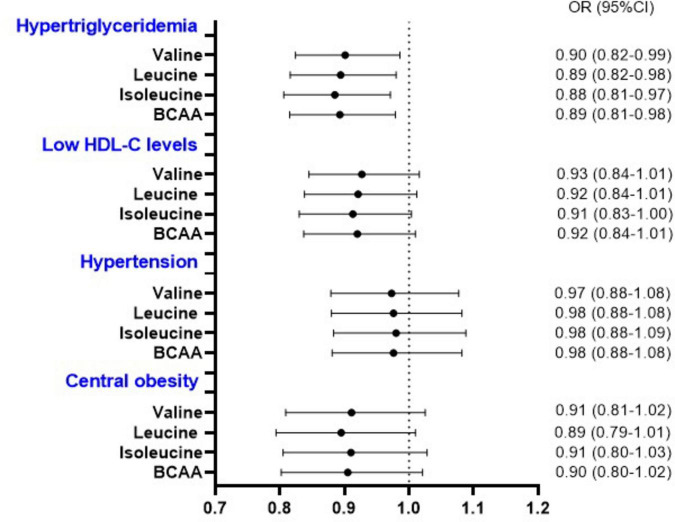
Odds of components of metabolic syndrome (other than hyperglycemia) per 1-S.D. increment in the energy-adjusted valine, leucine, isoleucine, and total BCAA intake. BCAA, branched-chain amino acids; CI, confidence interval; HDL-C, high-density lipoprotein cholesterol; OR, odds ratio. ORs and 95% CI were determined by multivariable logistic regression adjusted for age, sex, ethnicity, education, socioeconomic status, smoking status, alcohol intake, physical activity, energy, saturated fatty acids, protein, fiber intakes, and body mass index (Body mass index was not included in the model related to waist circumference).

We further assessed the association between BCAA intake and components of MetS. Multivariable analysis showed that subjects in the third and fourth quartiles of Val, Leu, Ile, and total BCAA intakes had lower odds of hyperglycemia (Table 4). Additionally, we detected a significant association between 1-S.D. increment of dietary Val, Leu, Ile, and total BCAA intake and the odds of hyperglycemia and hypertriglyceridemia. Nevertheless, no association was observed between the intake of these specific amino acids and other components of MetS (Figure 2). Furthermore, the animal-based to plant-based BCAA intake did not exhibit any significant association with the components of MetS (data not shown).

**TABLE 4:** Odds of metabolic syndrome according to the quartiles of energy-adjusted valine, leucine, isoleucine, and total BCAA intake.

Models	Quartile of energy-adjusted BCAA intake				BCAA intake per 1-S.D.	*P*
	Q1	Q2	Q3	Q4		
**Energy-adjusted valine**						
Event/Total	467/1215	454/1215	410/1215	446/1215		
OR (95%CI) *	1.00 (Ref.)	0.96 (0.81–1.13)	0.82 (0.69–0.96)	0.93 (0.79–1.09)	0.96 (0.90–1.01)	0.136
OR (95%CI) ^†^	1.00 (Ref.)	0.96 (0.81–1.15)	0.86 (0.71–1.03)	1.04 (0.86–1.26)	1.00 (0.93–1.07)	0.990
OR (95%CI) ^‡^	1.00 (Ref.)	0.88 (0.74–1.06)	0.74 (0.61–0.90)	0.77 (0.61–0.97)	0.84 (0.77–0.92)	**<0.001**
**Energy-adjusted leucine**						
Event/Total	469/1214	454/1216	417/1215	437/1215		
OR (95%CI)^ *^	1.00 (Ref.)	0.95 (0.80–1.11)	0.83 (0.70–0.98)	0.89 (0.76–1.05)	0.96 (0.90–1.01)	0.140
OR (95%CI) ^†^	1.00 (Ref.)	0.95 (0.80–1.14)	0.87 (0.72–1.05)	1.01 (0.83–1.22)	1.01 (0.94–1.08)	0.855
OR (95%CI) ^‡^	1.00 (Ref.)	0.87 (0.72–1.04)	0.73 (0.60–0.89)	0.72 (0.57–0.91)	0.84 (0.77–0.92)	**<0.001**
**Energy-adjusted isoleucine**						
Event/Total	467/1215	446/1214	420/1216	444/1215		
OR (95%CI) *	1.00 (Ref.)	0.93 (0.79–1.10)	0.84 (0.72–1.00)	0.92 (0.78–1.09)	0.96 (0.91–1.02)	0.194
OR (95%CI) ^†^	1.00 (Ref.)	0.95 (0.79–1.13)	0.88 (0.73–1.06)	1.02 (0.85–1.23)	1.00 (0.94–1.07)	0.933
OR (95%CI) ^‡^	1.00 (Ref.)	0.86 (0.72–1.03)	0.74 (0.61–0.90)	0.73 (0.58–0.92)	0.83 (0.75–0.91)	**<0.001**
**Energy-adjusted BCAA**						
Event/Total	471/1215	447/1215	416/1215	443/1215		
OR (95%CI) *	1.00 (Ref.)	0.92 (0.78–1.08)	0.82 (0.70–0.97)	0.91 (0.77–1.07)	0.96 (0.90–1.02)	0.154
OR (95%CI) ^†^	1.00 (Ref.)	0.93 (0.78–1.11)	0.86 (0.71–1.03)	1.01 (0.84–1.23)	1.00 (0.94–1.07)	0.932
OR (95%CI) ^‡^	1.00 (Ref.)	0.85 (0.71–1.02)	0.73 (0.60–0.89)	0.73 (0.58–0.93)	0.84 (0.76–0.92)	**<0.001**

BCAA, branched-chain amino acids; CI, confidence interval; OR, odds ratio. ORs and 95% CI were determined by multivariable logistic regression.

* Model 1: Crude and unadjusted,

^†^ Model 2: adjusted for age, sex, ethnicity, education, socioeconomic status, smoking status, alcohol intake, physical activity, body mass index, energy, saturated fatty acids, and fiber intakes,

^‡^ Model 3: additionally, adjusted for protein. Bold denotes significant change.

## 4 Discussion

In the current study, an inverse association between BCAA intake and the odds of MetS, hyperglycemia, and hypertriglyceridemia was identified, with odds reduction of 15%, 16%, and 11% per SD, respectively. Nonetheless, it is important to note that the observed associations were relatively weak due to the proximity of ORs to a value of one, which indicates triviality (28). Similar finding was found regarding Val, Leu, and Iles. We also detected no association between the animal-based to plant-based BCAA intake and odds of MetS and its components. This may suggest that focusing on increasing total BCAA intake could be more beneficial for preventing MetS than emphasizing the source of those BCAAs. However, further studies should be conducted in this regard.

Our findings are consistent with a previous cross-sectional study conducted on middle-aged Brazilian men, which demonstrated a negative association, independent of energy intake, physical activity, work position, and smoking status, between Leu and BCAA intake and the odds of MetS and hypertriglyceridemia (11). In a different cross-sectional study involving 8691 adults, no significant association was observed between BCAA intakes and odds of abdominal obesity (13). Nagata et al. (9) also conducted a cohort study and revealed that the consumption of a high percentage of BCAA as a part of total protein intake was significantly associated with a 43% reduction in the risk of diabetes in women, after controlling for demographic, anthropometric, lifestyle, medical, and dietary variables including total protein intake (9). However, in another cross-sectional study on female twins, higher BCAA intake was associated with lower insulin resistance, hypertension, and inflammation but not MetS, dyslipidemia, and central obesity. It is important to note that these associations were independent of genetics and several potential confounders, such as total protein intake (10). Higher BCAA intake also has been linked to a lower risk of cardiovascular diseases in patients with diabetes (12). In contrast, Isanejad et al. reported a weak positive relationship between dietary intake of BCAA and diabetes in postmenopausal women. This association remained significant even after making adjustments for total meat intake; however, the study did not account for total protein intake (29). Another study, conducted on three prospective cohorts - the Nurses’ Health Study, the Nurses’ Health Study II, and the Health Professionals Follow-up Study - showed a weak association between BCAA intake and the incidence of diabetes, which persisted despite attenuation after adjusting for meat and total protein intake (14). The presence of contradictory evidence may be attributed in part to heterogeneities in study designs, populations studied, the amounts and dietary sources of consumed BCAA, and the factors adjusted for. Notably, total protein intake has been linked to metabolic syndrome and diabetes (18, 30). Therefore, to ascertain an independent association between BCAA and MetS, not influenced by its role as a marker of protein consumption, we controlled the mentioned association for the total protein intake. It should be noted that several studies have found a weak association within the trivial range, with odds ratios ranging from 0.8 to 1.2. As such, further research is needed to determine the clinical applicability of these findings.

Several prior studies have examined the relationship between circulating BCAA and cardiometabolic risk factors. While some studies have reported a positive association between elevated plasma BCAA levels and insulin resistance, obesity, MetS, and cardiovascular diseases, the findings are not consistent across all studies. The regulation of circulating BCAA is influenced by the intestinal microbiome and metabolic dysfunction. However, there is a weak or null correlation between circulating BCAA levels and its dietary intakes (31). Further research is needed to elucidate the relationship between circulating and dietary BCAA intake, as well as the impact of protein quality on this association.

The precise mechanisms that underlie the relationship between BCAA consumption and MetS remain incompletely understood. It has been suggested that BCAA may reduce the accumulation of triglycerides in the liver and skeletal muscles, (32), thereby potentially mitigating the development of hypertriglyceridemia and insulin resistance (33, 34). This beneficial effect may be mediated by upregulation of peroxisome proliferator-activated receptor-alpha and uncoupling protein in these tissues (32). Furthermore, BCAA has been reported to attenuate hepatic lipid accumulation by reducing lipogenesis and increasing microbiota-mediated production of acetic acid (35). More studies should be performed to shed light on the other plausible mechanisms of BCAA as well as the potential effect of other amino acids in this regard.

Our research has several limitations that should be acknowledged. Firstly, the participants were recruited solely from an urban area in Kavar, a small county located in the Fars province in southwest Iran. Therefore, the generalizability of our findings to other populations or all Iranians may be limited. Secondly, the cross-sectional design of our study restricts our ability to establish causality and highlights the need for clinical trial. Lastly, despite adjusting for several confounding variables, there remains a possibility of residual confounders that may have influenced our results.

## 5 Conclusion

The results of this population-based cross-sectional study indicate that the total dietary intake of BCAA, as well as the individual intakes of Val, Leu, and Ile, were inversely associated with MetS, hyperglycemia, and hypertriglyceridemia. Additionally, no notable differences in this association were observed between animal-derived and plant-derived sources of BCAA.

## Data Availability

The original contributions presented in the study are included in the article/Supplementary material, further inquiries can be directed to the corresponding author.

## References

[1] Alberti K Eckel R Grundy S Zimmet P Cleeman J Donato K Harmonizing the metabolic syndrome: A joint interim statement of the international diabetes federation task force on epidemiology and prevention; national heart, lung, and blood institute; American heart association; world heart federation; international atherosclerosis society; and international association for the study of obesity. *Circulation.* (2009) 120:1640–5. 10.1161/CIRCULATIONAHA.109.192644 19805654

[2] Noubiap J Nansseu J Lontchi-Yimagou E Nkeck J Nyaga U Ngouo A Geographic distribution of metabolic syndrome and its components in the general adult population: A meta-analysis of global data from 28 million individuals. *Diabetes Res Clin Pract.* (2022) 188:109924. 10.1016/j.diabres.2022.109924 35584716

[3] Ranasinghe P Mathangasinghe Y Jayawardena R Hills A Misra A. Prevalence and trends of metabolic syndrome among adults in the Asia-pacific region: A systematic review. *BMC Public Health.* (2017) 17:101. 10.1186/s12889-017-4041-1 28109251 PMC5251315

[4] Shi T Wang B Natarajan S. The influence of metabolic syndrome in predicting mortality risk among US adults: Importance of metabolic syndrome even in adults with normal weight. *Prev Chron Dis.* (2020) 17:E36. 10.5888/pcd17.200020 32441641 PMC7279064

[5] Nilsson P Tuomilehto J Rydén L. The metabolic syndrome–what is it and how should it be managed? *Eur J Prev Cardiol.* (2019) 26:33–46. 10.1177/2047487319886404 31766917

[6] Institute of Medicine (US) Committee on Nutrition, Trauma, and the Brain, Erdman J Oria M Pillsbury L. *Nutrition and traumatic brain injury: Improving acute and subacute health outcomes in military personnel.* Washington, DC: National Academies Press (US) (2011). p. 8. 24983072

[7] Okekunle A Lee H Provido S Chung G Hong S Yu S Dietary branched-chain amino acids and odds of obesity among immigrant Filipino women: The Filipino women’s diet and health study (FiLWHEL). *BMC Public Health.* (2022) 22:1–9. 10.1186/s12889-022-12863-0 35382800 PMC8985351

[8] Qin L Xun P Bujnowski D Daviglus M Van Horn L Stamler J Higher branched-chain amino acid intake is associated with a lower prevalence of being overweight or obese in middle-aged East Asian and Western adults. *J Nutr.* (2011) 141:249–54. 21169225 10.3945/jn.110.128520 PMC3021443

[9] Nagata C Nakamura K Wada K Tsuji M Tamai Y Kawachi T. Branched-chain amino acid intake and the risk of diabetes in a Japanese community: The Takayama study. *Am J Epidemiol.* (2013) 178:1226–32. 10.1093/aje/kwt112 24008908

[10] Jennings A MacGregor A Pallister T Spector T Cassidy A. Associations between branched chain amino acid intake and biomarkers of adiposity and cardiometabolic health independent of genetic factors: A twin study. *Int J Cardiol.* (2016) 223:992–8. 10.1016/j.ijcard.2016.08.307 27591698 PMC5074005

[11] CocatePNataliAde OliveiraAAlfenasRHermsdorffH. Consumption of branched-chain amino acids is inversely associated with central obesity and cardiometabolic features in a population of Brazilian middle-aged men: Potential role of leucine intake. *J Nutr Health Aging.* (2015) 19:771–7. 10.1007/s12603-015-0521-0 26193862

[12] Zheng L Cai J Feng YH Su X Chen S-Y Liu J-Z The association between dietary branched-chain amino acids and the risk of cardiovascular diseases in Chinese patients with type 2 diabetes: A hospital-based case–control study. *Front Nutr.* (2022) 9:999189. 10.3389/fnut.2022.999189 36313094 PMC9614346

[13] Asoudeh F Salari-Moghaddam A Keshteli A Esmaillzadeh A Adibi P. Dietary intake of branched-chain amino acids in relation to general and abdominal obesity. *Eat Weight Disord.* (2022) 27:1303–11. 34268715 10.1007/s40519-021-01266-6

[14] Zheng Y Li Y Qi Q Hruby A Manson J Willett W Cumulative consumption of branched-chain amino acids and incidence of type 2 diabetes. *Int J Epidemiol.* (2016) 45:1482–92. 10.1093/ije/dyw143 27413102 PMC5100612

[15] Liu Y Zhang C Zhang Y Jiang X Liang Y Wang H Association between excessive dietary branched-chain amino acids intake and hypertension risk in Chinese population. *Nutrients.* (2022) 14:2582. 10.3390/nu14132582 35807761 PMC9268479

[16] Mirmiran P Teymoori F Asghari G Azizi F. Dietary intakes of branched chain amino acids and the incidence of hypertension: A population-based prospective cohort study. *Arch Iran Med.* (2019) 22:182–8. 31126176

[17] Yu L Zhu Q Li Y Song P Zhang J. Dietary branched-chain amino acids (BCAAs) and risk of dyslipidemia in a Chinese population. *Nutrients.* (2022) 14:1824. 10.3390/nu14091824 35565798 PMC9103899

[18] Hu W Sun L Gong Y Zhou Y Yang P Ye Z Relationship between branched-chain amino acids, metabolic syndrome, and cardiovascular risk profile in a Chinese population: A cross-sectional study. *Int J Endocrinol.* (2016) 2016:8173905. 10.1155/2016/8173905 27528871 PMC4977397

[19] Safarpour A Fattahi M Niknam R Tarkesh F Mohammadkarimi V Boogar S Alarm of non-communicable disease in Iran: Kavar cohort profile, baseline and 18-month follow up results from a prospective population-based study in urban area. *PLoS One.* (2022) 17:e0260227. 10.1371/journal.pone.0260227 35085244 PMC8794109

[20] Chalasani N Younossi Z Lavine J Diehl A Brunt E Cusi K The diagnosis and management of non-alcoholic fatty liver disease: Practice guideline by the American gastroenterological association, American association for the study of liver diseases, and American college of gastroenterology. *Gastroenterology.* (2012) 142:1592–609. 22656328 10.1053/j.gastro.2012.04.001

[21] Xun P Hou N Daviglus M Liu K Morris J Shikany J Fish oil, selenium and mercury in relation to incidence of hypertension: A 20-year follow-up study. *J Intern Med.* (2011) 270:175–86. 10.1111/j.1365-2796.2010.02338.x 21205024 PMC3070957

[22] Eghtesad S Mohammadi Z Shayanrad A Faramarzi E Joukar F Hamzeh B The PERSIAN cohort: Providing the evidence needed for healthcare reform. *Arch Iran Med.* (2017) 20:691–5. 29480734

[23] Eghtesad S Masoudi S Sharafkhah M Rashidkhani B Esmaeili-Nadimi A Najafi F Validity and reproducibility of the PERSIAN Cohort food frequency questionnaire: Assessment of major dietary patterns. *Nutr J.* (2024) 23:35. 10.1186/s12937-024-00938-0 38481332 PMC10935787

[24] Eghtesad S Hekmatdoost A Faramarzi E Homayounfar R Sharafkhah M Hakimi H Validity and reproducibility of a food frequency questionnaire assessing food group intake in the PERSIAN Cohort Study. *Front Nutr.* (2023) 10:1059870. 10.3389/fnut.2023.1059870 37599697 PMC10436288

[25] United States Department of Agriculture. *Food data central.* Washington, DC: United States Department of Agriculture (2021).

[26] Willett W Howe G Kushi L. Adjustment for total energy intake in epidemiologic studies. *Am J Clin Nutr.* (1997) 65:1220S–8S. 9094926 10.1093/ajcn/65.4.1220S

[27] Bifari F Nisoli E. Branched-chain amino acids differently modulate catabolic and anabolic states in mammals: A pharmacological point of view. *Br J Pharmacol.* (2017) 174:1366–77. 10.1111/bph.13624 27638647 PMC5429325

[28] Maher J Markey J Ebert-May D. The other half of the story: Effect size analysis in quantitative research. *CBE Life Sci Educ.* (2013) 12:345–51. 10.1187/cbe.13-04-0082 24006382 PMC3763001

[29] Isanejad M LaCroix A Thomson C Tinker L Larson J Qi Q Branched-chain amino acid, meat intake and risk of type 2 diabetes in the Women’s Health Initiative. *Br J Nutr.* (2017) 117:1523–30. 10.1017/S0007114517001568 28721839 PMC6450654

[30] Yuan S Ming-Wei L Qi-Qiang H Larsson S. Egg, cholesterol and protein intake and incident type 2 diabetes mellitus: Results of repeated measurements from a prospective cohort study. *Clin Nutr.* (2021) 40:4180–6. 10.1016/j.clnu.2021.01.041 33593662

[31] Zazpe I Ruiz-Canela M. Effect of branched-chain amino acid supplementation, dietary intake and circulating levels in cardiometabolic diseases: An updated review. *Curr Opin Clin Nutr Metab Care.* (2020) 23:35–50. 10.1097/MCO.0000000000000614 31688095

[32] Arakawa M Masaki T Nishimura J Seike M Yoshimatsu H. The effects of branched-chain amino acid granules on the accumulation of tissue triglycerides and uncoupling proteins in diet-induced obese mice. *Endocr J.* (2011) 58:161–70. 10.1507/endocrj.k10e-221 21372430

[33] Park S Seo Y. Excess accumulation of lipid impairs insulin sensitivity in skeletal muscle. *Int J Mol Sci.* (2020) 21:1949. 10.3390/ijms21061949 PMC7139950 32178449

[34] London A Lundsgaard A Kiens B Bojsen-Møller K. The role of hepatic fat accumulation in glucose and insulin homeostasis—dysregulation by the liver. *J Clin Med.* (2021) 10:390. 10.3390/jcm10030390 33498493 PMC7864173

[35] Iwao M Gotoh K Arakawa M Endo M Honda K Seike M Supplementation of branched-chain amino acids decreases fat accumulation in the liver through intestinal microbiota-mediated production of acetic acid. *Sci Rep.* (2020) 10:1–11. 10.1038/s41598-020-75542-3 33127939 PMC7603487

