# Supplementary material for: Intake of dietary branched-chain amino acids reduces odds of metabolic syndrome: a cross-sectional study on the PERSIAN Kavar cohort study
Source: Front Nutr. 2024 Oct 17;11:1403937. doi: 10.3389/fnut.2024.1403937 (PMC11525787; doi:10.3389/fnut.2024.1403937)
Supplement: Supplementary file 1 [file Table_1.docx]

**Supplementary Table 1. Comparison of amino acid intakes according to the metabolic syndrome status**

| **Variable** | **With metabolic syndrome** | **Without metabolic syndrome** | **P-value** |
| --- | --- | --- | --- |
| Dietary glycine intake (g/ day), mean ± SD | 1.75±0.71 | 1.78±0.71 | 0.057 |
| Dietary alanine intake (g/ day), mean ± SD | 2.19±0.85 | 2.23±0.86 | 0.059 |
| Dietary arginine intake (g/ day), mean ± SD | 2.68±1.03 | 2.74±1.04 | **0.031** |
| Dietary aspartic acid intake (g/ day), mean ± SD | 4.64±1.72 | 1.72±1.76 | 0.145 |
| Dietary glutamic acid intake (g/ day), mean ± SD | 8.09±2.88 | 8.25±2.95 | 0.069 |
| Dietary cysteine intake (g/ day), mean ± SD | 0.61±0.22 | 0.63±0.23 | **0.004** |
| Dietary histidine intake (g/ day), mean ± SD | 1.13±0.45 | 1.15±0.46 | 0.060 |
| Dietary lysine intake (g/ day), mean ± SD | 2.87±1.19 | 2.93±1.20 | 0.109 |
| Dietary methionine intake (g/ day), mean ± SD | 0.94±0.38 | 0.96±0.38 | 0.062 |
| Dietary phenylalanine intake (g/ day), mean ± SD | 1.93±0.72 | 1.98±0.73 | **0.014** |
| Dietary proline intake (g/ day), mean ± SD | 2.31±0.88 | 2.37±0.92 | **0.015** |
| Dietary serine intake (g/ day), mean ± SD | 2.06±0.78 | 2.11±0.76 | **0.010** |
| Dietary threonine intake (g/ day), mean ± SD | 1.70±0.66 | 1.74±0.67 | **0.035** |
| Dietary tryptophan intake (g/ day), mean ± SD | 0.48±0.18 | 0.50±0.19 | **0.018** |
| Dietary tyrosine intake (g/ day), mean ± SD | 1.43±0.55 | 1.47±0.56 | **0.040** |

Abbreviations: SD: standard deviation.

Between-group differences were assessed using the independent sample t-test.

P<0.05 was considered significant.
